# Novel fluorescent-based reporter cell line engineered for monitoring homologous recombination events

**DOI:** 10.1371/journal.pone.0237413

**Published:** 2021-04-30

**Authors:** Alejandra Bernardi, Dino Gobelli, Julia Serna, Paulina Nawrocka, Gabriel March-Rosselló, Antonio Orduña, Piotr Kozlowski, María Simarro, Miguel A. de la Fuente

**Affiliations:** 1 Institute of Biomedicine and Molecular Genetics (IBGM) of Valladolid, Valladolid, Spain; 2 Department of Molecular Genetics, Institute of Bioorganic Chemistry, Polish Academy of Sciences, Poznan, Poland; 3 Division of Microbiology, Hospital Clínico of Valladolid, Valladolid, Spain; 4 Microbiology Department, University of Valladolid, Valladolid, Spain; 5 Department of Nursing–“Grupo de Investigación en Cuidados de Enfermería” GICE, University of Valladolid, Valladolid, Spain; 6 Department of Cell Biology, Histology and Pharmacology, University of Valladolid, Valladolid, Spain; University of Miami School of Medicine, UNITED STATES

## Abstract

Homologous recombination (HR) faithfully restores DNA double-strand breaks. Defects in this HR repair pathway are associated with cancer predisposition. In genetic engineering, HR has been used extensively to study gene function and it represents an ideal method of gene therapy for single gene disorders. Here, we present a novel assay to measure HR in living cells. The HR substrate consisted of a non-fluorescent 3’ truncated form of the eGFP gene and was integrated into the AAVS1 locus, known as a safe harbor. The donor DNA template comprised a 5’ truncated eGFP copy and was delivered via AAV particles. HR mediated repair restored full-length eGFP coding sequence, resulting in eGFP+ cells. The utility of our assay in quantifying HR events was validated by exploring the impact of the overexpression of HR promoters and the siRNA-mediated silencing of genes known to play a role in DNA repair on the frequency of HR. We conclude that this novel assay represents a useful tool to further investigate the mechanisms that control HR and test continually emerging tools for HR-mediated genome editing.

## Introduction

DNA double-strand breaks (DSBs) are among the most serious types of DNA damage in cells and can lead to genetic instability and tumorigenesis [1]. DSB may be induced by exogenous genotoxic insults, such as ionizing radiation, but also occur spontaneously in various cellular processes including DNA replication and V(D)J recombination. There are two major pathways for DSB repair: non-homologous end joining (NHEJ) and homologous recombination (HR). NHEJ directly ligates the two broken ends of a DSB and is accessible throughout the cell cycle. In contrast, HR is an error-free repair mechanism that primarily uses the intact sister chromatid as a template for repair and predominates in S-phase cells [2].

The physiological importance of HR is underscored by the association of cancer predisposition and developmental defects with mutations in HR genes. The tumor suppressors BRCA1 and BRCA2, key players at different stages of HR, are frequently mutated in familial breast and ovarian cancers [3, 4]. Other HR proteins, including PALB2 and RAD51 paralogs, have also been identified as tumor suppressors [3, 4]. On the other hand, tumor cells with defective HR repair show increased sensitivity to chemotherapeutic reagents that act via the induction of DNA damage, including platinum-containing agents and PARP inhibitors [3, 4]. These observations suggest that HR-proficient tumor cells might be sensitized to chemotherapeutics if HR repair could be therapeutically inactivated.

In genetic engineering, HR represents a powerful tool to precisely manipulate the genome for experimental uses. Its use revolutionized the genetic approach to study biological processes in mice by the late 1980s with the generation of the first gene targeted knockout mice [5]. Since then, thousands of genes have been modified in mouse embryonic stem cells by HR with gene-targeting vectors. However, the low efficiency of HR in vertebrate somatic cells limits the utility of this approach [6]. Following the discovery that induction of a DSB increases the frequency of homology directed repair (HDR) by several orders of magnitude, targeted nucleases have emerged as the method of choice for improving the efficiency of HDR-mediated genetic alterations [7]. ZFNs (zinc finger nucleases), TALENs (transcription activator-like effector nucleases) and CRISPR/Cas9 (clustered regularly interspaced short palindromic repeats/CRISPR-associated system 9) are all engineered endonucleases that can introduce DSBs at desired locations in the genome. Once a targeted DSB has been made, HDR may reconstruct the cleaved DNA using an exogenous DNA template [7].

Various cell-based assays have been developed for the accurate measurement of HR events. The use of fluorescence has proved to be an effective approach for detecting HR and the most commonly used assay is the direct repeat green fluorescent protein (DR-GFP) assay [8, 9]. The DR-GFP reporter consists of two tandem, inactive copies of the GFP gene, which can be integrated into the cellular genome or expressed transiently. The first GFP copy (SceGFP) has a promoter and contains the I-SceI restriction site with an in-frame stop codon and the second copy (iGFP) harbours truncations at both ends [8, 9]. After cleavage within SceGFP by I-SceI or a Cas9 nuclease, HR uses iGFP as a template to restore the GFP gene to its functional form. Then, GFP fluorescence can be measured using flow cytometry [8, 9].

Here, we report the development of a novel fluorescence-based assay for evaluating cellular HR activity. As opposed to the DR-GFP assay, in our system HR reconstitutes the full-length coding sequence of the enhanced green fluorescent protein (eGFP), which fluoresces much more intensely than GFP, thus facilitating the detection of HR events. Our assay also differs from the DR-GFP assay in that our two non-functional copies of eGFP are not in tandem. Rather, a single copy of the HR substrate is integrated as a single copy into the AAVS1 safe harbor locus and the template donor is delivered exogenously via AAV particles, mimicking the scenario of the HR-mediated correction of a monogenic disorder. Following this procedure, we have detected changes in HR rates resulting from the altered expression of DNA repair genes, similar to those previously reported. We provide a well-characterized tool that will help to further investigate the mechanisms that control HR and analyze the effects of exogenous manipulations, with i.e., drugs, miRNAs, and genes on HR mediated correction.

## Materials and methods

### Cells

Human colon cancer cell line HCT116 was obtained from the American Type Culture Collection (Manassas, VA). Human embryonic kidney cell line HEK-293 optimized for the packaging of AAV virions (AAV-293) was obtained from Agilent Technologies (Santa Clara, CA). Both cell lines were handled according to each manufacturer’s recommendations.

### Plasmids construction

The sequences of the primers used in this study are shown in **S1 Table**. The pAAV-MCS-eGFPΔ3’ plasmid (also named as peGFPΔ3’) used to generate the HR reporter cell line was obtained through the following steps. Firstly, a fragment containing the 5’AAVS1 region homology arm (832 bp) followed by a puromycin cassette (1014 bp) was amplified from pAAV-CAGGS-eGFP plasmid with primers 1F and 1R. pAAV-CAGGS-eGFP plasmid was a gift from Rudolf Jaenisch (Addgene plasmid # 22212; http://n2t.net/addgene:22212; RRID:Addgene_22212) [10]. The puromycin cassette (SA-T2A-Puro-pA) is promoterless and contains a splice acceptor site followed by the coding sequence of the T2A peptide, the puromycin resistance gene and a polyadenylation signal. Secondly, a 1290 bp fragment containing human cytomegalovirus (CMV) immediate early (IE) promoter and eGFP lacking the last 73 bp was amplified from peGFP-C1 plasmid (Clontech) with primers 2F and 2R. Thirdly, the 3’AAVS1 region homology arm (1300 bp) was amplified from HCT116 genomic DNA using 3F and 3R. The three fragments were digested and cloned into the NotI site of pAAV-MCS (Stratagene). With respect to the designation of the primers used in this work, F stands for forward, and R for reverse.

The HR donor plasmid, pAAV-MCS-eGFPΔ5’ (also named as peGFPΔ5’), was obtained through the following steps. Firstly, a 929 bp fragment containing eGFP lacking the first 38 bp at 5’ end was amplified from peGFP-N1 plasmid (Clontech) with primers 4F and 4R. Secondly, an EcoRI-SalI fragment (1582 bp) containing a blasticidin resistance cassette flanked by loxP sequences was obtained from a pBluescript II based plasmid previously generated in our lab. Thirdly, the 3’AAVS1 region homology arm (1300 bp) was obtained as described for pAAV-MCS-eGFPΔ3’ plasmid. The three fragments were digested and cloned into the NotI site of pAAV-MCS (Stratagene). AAV2 particles were produced by co-transfecting 293 c18 cells (ATCC CRL-10852) with the pAAV-MCS- eGFPΔ5’ donor plasmid, pHelper and pAAV-RC (at a 1:1:1 ratio). Three days later cells were harvested and AAV were released by 4 freeze-thaw cycles. Viral titers were determined by SybrGreen based real time qPCR using ITR_F and ITR_R primers as previously described [11]. pHelper and pAAV-RC plasmids were obtained from Stratagene.

The RAD52 gene of *Saccharomyces cerevisiae* (ScRAD52) was cloned into the expression vector pET15b (Novagen) for His-tagged production of TAT-NLS-RAD52 where TAT peptide (GRKKRRQRRR) promotes cell permeability and NLS peptide (KKKRKV) is a nuclear localization signal. Wild-type RAD52 sequence was amplified by PCR from the genomic DNA of *Saccharomyces cerevisiae* with primers ScRAD52_F1 and ScRAD52_R1 and cloned into HindIII/XhoI digested pTriEx-HTNC vector immediately downstream the His-TAT-NLS sequence. pTriEx-HTNC was a gift from Klaus Rajewsky (Addgene plasmid # 13763) [12]. The resultant construct was digested with NcoI and XhoI and the His-TAT-NLS-ScRAD52 fragment was cloned into NcoI/XhoI digested pET15b, thus obtaining pET15b-TAT-NLS-ScRAD52. pET15b-TAT-NLS-ScRAD52 was transformed into BL21 (DE3) and the selected bacteria were grown. His-TAT-NLS-ScRAD52 expression was induced with 1 mM IPTG for 3 h and the recombinant protein was purified using Nickel-Sepharose beads from the soluble fraction of the bacterial extracts. Recombinant protein was stored in a solution containing 50% (v/v) glycerol, 20 mM HEPES (pH = 7.4) and 500 mM NaCl. Several concentrations of TAT-NLS-ScRAD52 ranging from 0.02 to 2 μM were tested for their capacity to increase the HR frequency. The maximum frequencies were obtained with concentrations equal to or greater than 0.2 μM, and a significant level of cytotoxicity was observed only at concentrations higher than 1.8 μM. The TAT-NLS-ScRAD52 experiments shown in this work were performed using the fusion protein at a concentration of 0.2 μM.

DNA fragments encoding ScRAD52, RAD51, and RAD52 Flag-tagged at the N-termini were generated by PCR and cloned into mammalian expression vector pcDNA3 (Invitrogen). The Flag sequence was added to the forward primers. The restriction sites used in the cloning step are shown in **S1 Table**. ScRAD52 was amplified with the primer pair ScRAD52_F2/ ScRAD52_R2 using pET15b-TAT-NLS-ScRAD52 plasmid as template. Human RAD51 was amplified from the plasmid CMV-hRad51 using primers hRAD51_F/hRAD51_R. CMV-hRad51 was a gift from David Liu (Addgene plasmid # 125570; http://n2t.net/addgene:125570; RRID:Addgene_125570) [13]. Human RAD52 was amplified from the plasmid pMM1413-SUMO-RAD52 using primers hRAD52_F/hRAD52_R. pMM1413-SUMO-RAD52 was a gift from Mauro Modesti (Cancer Research Center of Marseille). The resultant constructs were named pScRAD52, phRAD51, and phRAD52. The plasmid encoding for Flag-PALB2 was identified as phPALB2 in this work and corresponds to the expression plasmid pDEST-FRT/T0-Flag-PALB2. pDEST-FRT/T0-Flag-PALB2 was a gift from Daniel Durocher (Addgene plasmid # 71114; http://n2t.net/addgene:71114; RRID:Addgene_71114) [14]. The constructs were transfected into the reporter cell line when indicated, and the expression of the Flag-tagged HR promoters was analyzed by Western Blot using mouse monoclonal antibodies against Flag peptide (clone M2, Sigma-Aldrich) and β-actin (AC-40; Sigma-Aldrich) as the loading control.

### Generation of the HCT116-eGFPΔ3’ reporter cell line and HR-mediated rescue of eGFP expression

HCT116 cells were nucleofected with pAAV-MCS-eGFPΔ3’ plasmid and AAVS1 ZFN mRNA (Sigma-Aldrich). AAVS1 ZFN mRNA encodes a pair of ZFNs that target the genomic integration site of AAVS1. Targeted integration of pAAV-MCS-eGFPΔ3’ in puromycin-resistant individual clones was verified by PCR using the following primers: P1F and P1R for analysis of 5’-arm recombination; P2F and P2R for analysis of 3’-arm recombination. Homo- and heterozygosity of the eGFPΔ3’ transgene at the AVVS1 locus was explored by PCR using primers P1F and P2R located outside the homology arms. The single copy integration of eGFPΔ3’ into the AAVS1 locus was verified by Multiplex Ligation-Dependent Probe Amplification (MLPA) and droplet digital PCR (ddPCR) (see below). The resultant cell line was named HCT116-eGFPΔ3’. HCT116-eGFPΔ3’ cells were transduced with AAV particles containing pAAV-MCS-eGFPΔ5’ donor plasmid (MOI of 10^3^). HR leads to reconstitution of full-length eGFP coding sequence and the appearance of green fluorescent cells 48 hours post-transduction. Individual clones were obtained by limiting dilution in the presence of blasticidin (5 μg/ml) and were analyzed by PCR with primers P3F and P3R. The following primers against human SDHA were used for the genomic DNA loading control PCR: SDHA_F and SDHA_R. The restored full length eGFP cassette was also sequenced and its expression analyzed by Western Blot using mouse monoclonal antibodies against GFP (clone B34, Biolegend) and β-actin (AC-40; Sigma-Aldrich) as the loading control.

### Multiplex ligation-dependent probe amplification (MLPA)

MLPA reactions were performed according to the manufacturer’s general recommendations (MRC-Holland) with the use of the probes designed and generated according to the strategy developed [15] and described in detail before [16]. Briefly, 100 ng DNA in 5 μl from parental HCT116 and reporter cell line were denaturated for 5 min at 98°C, cooled to room temperature and mixed with 1.5 μl of probes mix (containing 1.5 fmol of each probe), and 1.5 μl of SALSA hybridization buffer. The reaction was then denatured at 95°C for 1 min and probes were hybridized to their respective targets at 60°C for 16–20 h. After the addition of 32 μl of ligation mixture, the hybridized probes were ligated together at 54°C for 15 min. After heat inactivation, ligation reaction was cooled to room temperature, mixed with 10 μl of PCR mixture (polymerase, dNTPs, and universal primers, one of which was labeled with fluorescein) and subjected to PCR amplification for 35 cycles. The MLPA products were subsequently diluted 20x in HiDi formamide containing GS Liz600, which was used as a DNA size standard and separated by capillary electrophoresis (POP7 polymer) using an ABI Prism 3130XL apparatus (Applied Biosystems). The electropherograms were visualized and analyzed using GeneMarker software v1.91 (2.4.0). The eGFP-specific MLPA probes and 7 control MLPA probes are listed in **S2 Table**. For each probe, the sequences of the 5’ and 3’ half-probes, universal sequences to which PCR primers are targeted, the “stuffer” sequence, and the target specific sequence are provided. Details on gene loci and chromosome locations for control probes are also provided.

### Droplet digital PCR (ddPCR)

The ddPCR was performed using the QX200 system and EvaGreen Supermix (BIO-RAD), according to the manufacturer’s general recommendations, as generally described [17] and used before [18]. To determine the exact copy number of the eGFP transgene in the reporter cell line, we designed two test-amplicons entirely located in the transgene (eGFP1 and eGFP2) and two control-amplicons [C1 (up) and C2 (down)], located upstream (2 Kb) and downstream (overlapping exon 2 of *PPP1R12C*) of the transgene site (AAVS1), respectively. The following sets of primers were used: (i) T1F and T1R for test amplicon eGFP1 (187 bp in length); (ii) T2F and T2R for test-amplicon eFGP2 (163 bp in length); (iii) C1F and C1R for control-amplicon C1 (up) (170 bp in length); and (iv) C2F and C2R for control-amplicon C2 (down) (238 bp in length). Of note, genomic DNA from the reporter cell line was digested with the *CviQ*I restriction enzyme (New England Biolabs) prior to ddPCR. *CviQ*I was chosen because it cuts the transgene between the sequences corresponding to amplicons eGFP1 and eGFP2, which prevents tandemly repeated transgenes from being incorrectly identified as a single copy. Briefly, ddPCR procedure was as follow: the PCR mixture containing 10 μl of EvaGreen Supermix (Bio-Rad), 1 μl of 4 μM forward primer, 1 μl of 4 μM reverse primer, 4 μl (80 ng) of DNA from the tested cell line and 6 μl of water was partitioned into 20,000 droplets with the use of a QX200 ddPCR droplet generator (Bio-Rad). The generated droplets were transferred to a 96-well plate and amplified in a T100 Thermal Cycler (BioRad) under the following conditions: 5 min at 95°C, followed by 40 cycles of 30 s at 95°C, 30 s at 60°C, and 45 s at 72°C, followed by 2 min at 72°C, 5 min at 4°C, 5 min at 90°C for enzyme inactivation and holding at 12°C. The amplified products were analyzed using a QX200 droplet reader (Bio-Rad), and the number of positive and negative droplets were counted using the QuantaSoft version 1.7.4.019 software (Bio-Rad). The copy number of eGFP was calculated based on the number of positive droplets in eGFP-specific PCR reactions (either eGFP1 and eGFP2) normalized against the average number of positive droplets in control reactions [C1 (up) and C2 (down)]. Control amplicons were selected in diploid regions and their copy numbers set to 2.

### HR assay and screening protocol

In all the assays, HCT116-eGFPΔ3’ cells were sequentially treated with: (i) the selected siRNA oligoes, or expression plasmids encoding HR promoters, (ii) a TALEN pair designed to facilitate HR, and (iii) AAV particles containing the donor plasmid (**S1 Fig**). In brief, HCT116-eGFPΔ3’ were plated on 12 well plates at 125,000 cells/well. Forty-eight hours later, cells were transfected with siRNAs using TransIT-X2 (Mirus Bio). The siRNAs used in this work are listed in **S3 Table**. Silencer Select highly potent and chemically modified siRNAs were used at a final concentration of 5 nM, whereas classical siRNAs were used at a final concentration of 40 nM. Alternatively, cells were transfected with 400 ng of the expression plasmids encoding HR promoters using TransIT-X2. Twenty-four hours after the first treatment, cells were transfected with a TALEN pair designed to facilitate HR. The TALEN pair (named eGFP) targeted a region within the AAVS1 locus adjacent to the 3’end of eGFPΔ3’ in the HCT116-eGFPΔ3’ cell line. We used the fuzznuc software provided by the EMBOSS package to identify the targeting sequences: 5’-TGCCAGAACCTCTAAGGTTT-3’ (sense component) and 5’-TCCCTCCCAGGATCCTCTCT-3’ (antisense component). TALEN expression vectors were constructed using the LIC (ligation-independent cloning) TAL Effector Assembly Kit (Addgene #1000000023). The kit provides a TALE repeat unit library of 2-mer fragments that were assembled into an expression ready TALEN construct in 2 hierarchical assembly steps. Six hours after the transfection of HCT116-eGFPΔ3’ cells with 1 μg of each of the TALEN expression plasmids, cells were transduced with AAV particles containing pAAV-MCS-eGFPΔ5’ donor plasmid (MOI of 10^3^). When indicated, reporter cells were treated with TAT-NLS-ScRAD52 (at a final concentration of 0.2 μM) 5 hours after the transfection of the TALEN plasmids and 1 hour before the infection with the AAV particles. After 24 hours, medium was replaced with fresh medium, and after the next 24 hours (48 hours post-transduction), the recombination frequency was determined as the percentage of cells expressing eGFP protein following flow cytometry analysis using a Beckman Coulter Gallios (Beckman Coulter). Data were analyzed using Kaluza software (Beckman Coulter) and were subsequently corrected for transfection and transduction efficiencies **(S2 Fig)**. Dead cells and debris were excluded based on scatter signals and propidium iodide fluorescence. When indicated, cells were fixed, stained with Hoechst 33342 (5 μg/ml) and imaged using a Nikon Eclipse 90i fluorescent microscope coupled to a Nikon DS-Ri1 CCD camera.

The transfection efficiency of the eGFP-TALEN plasmids and siRNAs using TransIT- was >85% in all the HR experiments shown in this work. Transfection efficiency was measured in parallel to HR experiments by cotransfecting HCT116-eGFPΔ3’ with either eGFP-TALEN plasmids, expression plasmids or siRNAs under the same conditions as in the HR experiments but in combination with 1 μg of peGFP-C1. Twenty-four hours later, cells were analyzed for eGFP by flow cytometry analysis and the percentage of eGFP positive (eGFP+) cells was determined to estimate the transfection efficiency. The efficacy of the siRNAs in gene silencing was validated by a SYBR Green based real-time PCR assay using the primers listed in **S1 Table**. HPRT1 and β-actin were used as housekeeping genes. All siRNAs tested showed over 70% target knockdown at 48 hours post-transfection (**S3 Fig**).

Transduction efficiency of AAV2 was tested using particles carrying the entire eGFP gene. AAV2 particles were prepared in 293 c18 cells using the plasmids pAAV-MCS-eGFP, pHelper and pAAV-RC (at a 1:1:1 ratio), as described above. HCT116 were transduced with different doses (from MOI 10 to 10^4^). The highest transduction efficiency (measured as frequency of eGFP+ cells) achieved with AAV2 in HCT116 was 10±1.5% (mean ± SEM) at MOI 10^3^, and corroborate previous findings [19].

### Statistics

All analyses were performed using Prism software (GraphPad). Data are expressed as mean ± SD and were analyzed by using the unpaired Student’s t-test. In all graphs, * p < 0.05, ** p < 0.01, *** p < 0.001.

## Results

### Development of a novel eGFP-based HR reporter system

In our assay, HR leads to the restoration of the full-length eGFP coding sequence from two different truncated eGFP copies. eGFP is a variant of the wild-type GFP with higher-intensity emission than GFP [20], thus facilitating the detection of HR events. In order to generate the two truncated eGFP copies, DNA sequences for amino acid residues shown to be essential for eGFP fluorescence [21] were deleted, such that the coding sequence lacked 72 bp at the 3’ end in the eGFPΔ3’ copy, and 42 bp at the 5’ end in eGFPΔ5’ (including the start codon). The assay was designed for the fragment eGFPΔ3’ (the recombination substrate) to be integrated in the genome, while eGFPΔ5’ (the donor template) is delivered exogenously via AAV particles.

We optimized the assay system in the human colon cancer cell line HCT116, which is highly proficient in gene targeting [22, 23] and has been widely used to study the molecular mechanisms of HR [24–26]. To avoid position effects, we aimed to integrate eGFPΔ3’ into the adeno-associated virus integration site 1 (AAVS1) within the intron 1 of PPP1R12C on human chromosome 19. We chose the AAVS1 locus as the target because it is considered a safe harbor locus for integrating transgenes, since it is constitutively expressed across a variety of cell types, including HCT116 cells, and biallelic disruption of PPP1R12C results in no discernible phenotype [27].

**Fig 1:**
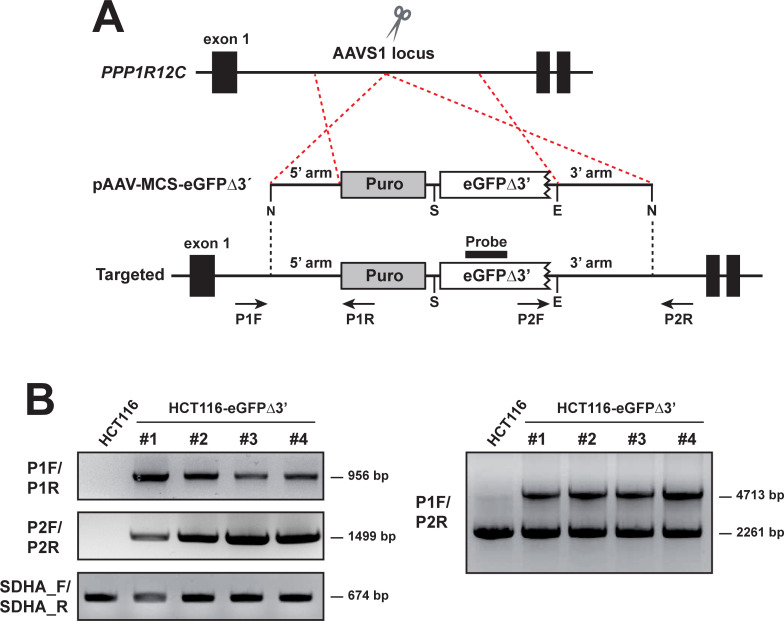
Generation of the HCT116-eGFPΔ3’ reporter cell line. (A) Schematic illustration of AAVS1 gene targeting with pAAV-MCS-eGFPΔ3’ plasmid. Puro, puromycin-polyA promotorless cassette; N, NotI; S, SalI; E, EcoRI. Exons are indicated by black boxes. (B) Representative PCR analysis for site-specific integration with the primer pairs P1F/P1R (5’ arm), P2F/P2R (3’ arm), and hSDHA_F/hSDHA_R (loading control). (C) Representative PCR analysis for the homo/heterozygosity targeting of the AAVS1 locus with the primer pair P1F/P2R. Positions of the primers used for screening are designated by arrows in panel A.

The targeting construct (peGFPΔ3’) contained a left and right homology arm to the AAVS1 genomic integration site flanking the eGFPΔ3’ transgene and a puromycin cassette. The targeting construct design strategy is shown in **Fig 1A**. The eGFPΔ3’ transgene was under the control of the CMV promoter. The puromycin selection cassette was preceded by a splice acceptor site. Therefore, its expression relied on splicing and was driven by the endogenous PPP1R12C promoter. Finally, HCT116 were nucleofected with the targeting construct peGFPΔ3’ and a well-validated pair of ZFNs engineered to target the AAVS1 locus and increase the targeting rate.

Puromycin-resistant clones were screened for successful integration of the transgene by PCR analysis of genomic DNA using different combinations of primers located in the transgene and outside the 5’ and 3’ homology arms as illustrated in **Fig 1A** and **1B** shows representative results of PCR analysis of 4 correctly targeted clones which were named HCT116-eGFPΔ3’ followed by a serial number. PCR using primers outside the arms showed all clones were heterozygotes for the eGFPΔ3’ transgene. We randomly selected the cell clone HCT116-eGFPΔ3’ #1 for further experiments.

**Fig 2:**
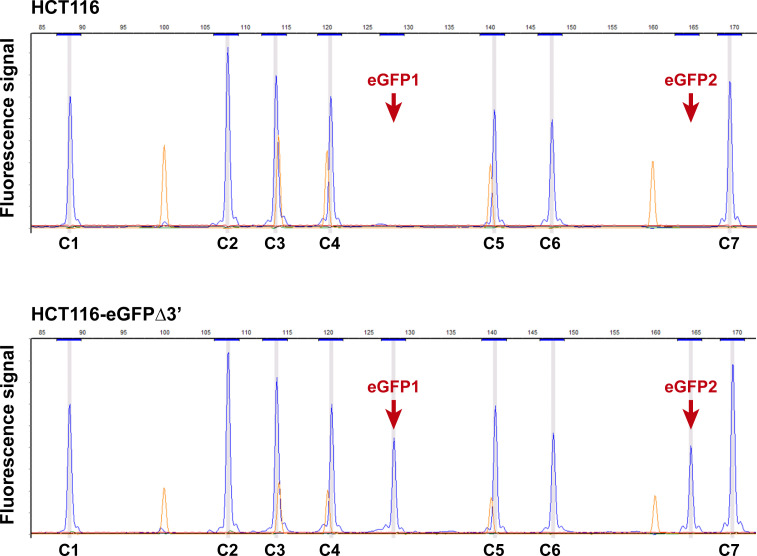
MLPA analysis for eGFPΔ3’. Electropherograms from parental HCT116 cells and the HCT116-eGFPΔ3’ reporter cell line. Blue peaks represent signals of MLPA probes used for the indicated genes. The red arrows indicate signals corresponding to eGFP1 and eGFP2 probes. The eGFP-specific MLPA probes and 7 control MLPA probes are listed in **S2 Table**. Orange peaks represent fragments of GS Liz600 DNA size standard.

**Fig 3:**
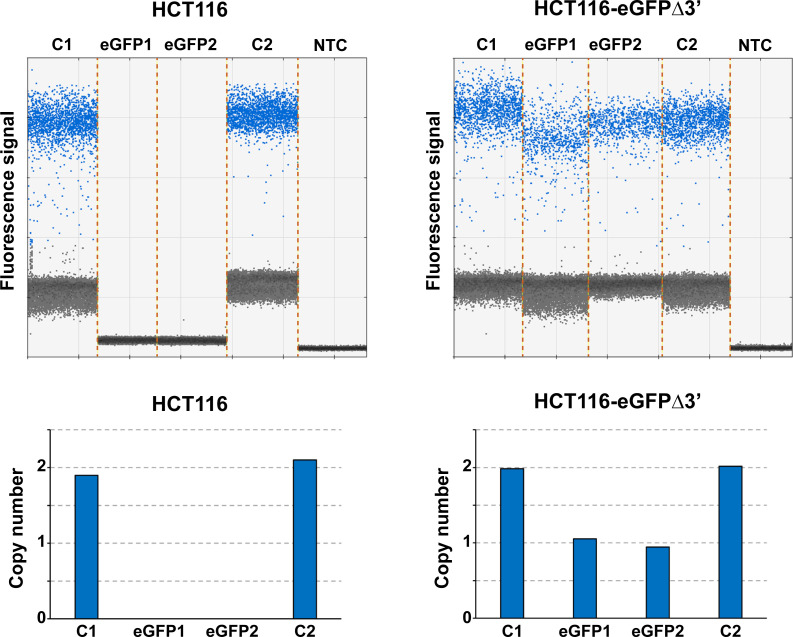
Determination of eGFPΔ3’ copy number by ddPCR. Upper graphs depict droplets fluorescence intensity for the indicated reactions using genomic DNA from either parental HCT116 cells or the HCT116-c reporter cell line. NTC stands for “no template control”. Blue specks denote droplets classified as positive. Black specks denote droplets classified as negative. The bar-plots below show eGFPΔ3’copy number values in parental HCT116 cells or the HCT116-eGFPΔ3’ reporter cell line. Control amplicons were selected in diploid regions and the copy number was set to 2.

The presence of the transgene eGFPΔ3’ in the reporter cell line was confirmed by MLPA using two pairs of probes specific for eGFP (eGFP1 and eGFP2) and seven probes specific for diploid control regions (C1 to C7). **Fig 2** shows electropherograms of MLPA reactions performed on genomic DNA extracted from parental HCT116 and HCT116-eGFPΔ3’ #1 cell lines. eGFP-specific signals were present in the HCT116-eGFPΔ3’ #1 electropherogram (red arrowheads), but not in the parental HCT116 electropherogram, which confirmed the presence of the transgene in the HCT116-eGFPΔ3’ #1 cells. The peak heights for eGFP were approximately half those of the control probes suggesting that HCT116-eGFPΔ3’ #1 cells were heterozygous for the transgene. Next, ddPCR was performed to precisely determine the eGFPΔ3’ gene copy number and rule out integration at other off-target sites. Pairs of primers were designed to amplify two test-amplicons (eGFP1 and eGFP2) located entirely within the eGFPΔ3’ transgene and two control-amplicons (C1 and C2) specific for the diploid flanking regions. Of note, genomic DNA from the reporter cell line HCT116-eGFPΔ3’ #1 was digested with the *CviQ*I restriction enzyme prior to ddPCR. *CviQ*I was chosen because it cuts the transgene between the sequences corresponding to amplicons eGFP1 and eGFP2, thus preventing tandemly repeated transgenes from being misidentified as a single copy. As shown in **Fig 3**, the fluorescence intensity of the HCT116-eGFPΔ3’ #1 DNA droplets when eGFP1 and eGFP2 assays were used was half of that obtained with the control assays, indicating the HCT116-eGFPΔ3’ #1 reporter cell line contains a single copy of the eGFPΔ3’ transgene.

**Fig 4:**
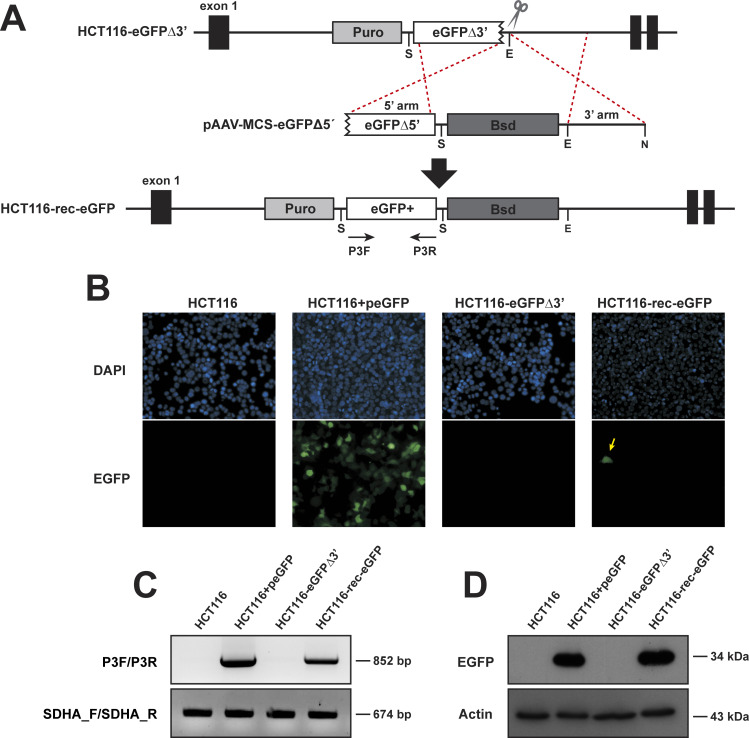
HR-mediated reconstitution of full-length eGFP. (A) Schematic illustration of HR-mediated eGFP reconstitution in the HCT116-eGFPΔ3’ reporter cell line after infection with AAV particles containing donor plasmid pAAV-MCS-eGFPΔ5’. Resultant eGFP+ cells are designated HCT116-rec-eGFP. (B) Fluorescent images of the indicated cell lines were captured using a 20x objective. The eGFP signal is shown in green and DAPI-stained nuclei are shown in blue. Parental HCT116+peGFP corresponds to wild type HCT116 transfected with a plasmid containing eGFP. The yellow arrow in the right panel depicts a HCT116-rec-eGFP cell. (C) PCR analysis for eGFP reconstitution with primer pairs P3F/P3R and hSDHA_F/hSDHA_R (for loading control). DNA was extracted from the indicated cell lines. HCT116-rec-eGFP refers to a single cell-derived clone. (D) Western blot analysis of total cell extracts from the same cells as in C using a polyclonal antibody against eGFP or β-actin (loading control).

Next, we produced AAV particles harboring the HR donor template that contained a truncated eGFP lacking the first 42 bp, a blasticidin cassette and the 3’AAVS1 homology arm (pAAV-MCS-eGFPΔ5’, **Fig 4A**). AAV particles were added onto HCT116-eGFPΔ3’ #1 cells at a MOI of 10^3^ viral particles/cell. Two days after transduction, eGFP+ cells (also named HCT116-rec-eGFP) were detected (**Fig 4B)**. The frequency of recombination was 4.3x10^-3^ ± 1.6x10^-3^. To formally determine whether or not fluorescence cells had undergone HR, we isolated fluorescent cells to assess if they harbored full-length eGFP coding sequences. Single HCT116-rec-eGFP cell clones were obtained by limiting dilution and then expanded to clonal cell lines in the presence of blasticidin. HR-induced restoration of an intact eGFP gene in fluorescent cells was assessed by PCR (**Fig 4C**) and sequencing. Western blot analysis confirmed the rescue of eGFP expression in HCT116-rec-eGFP cells (**Fig 4D**). The absence of a truncated form in HCT116-eGFPΔ3’ #1 cells is commented in the discussion section.

### Validation of the HR system

Next, we evaluated the ability of our assay to identify factors affecting HR. To measure HR activity, eGFP+ cells were counted by flow cytometry and the raw data was subsequently corrected for transfection and transduction efficiencies as described in Materials and Methods and in **S2 Fig**.

In all the experiments that will be described below, HCT116-eGFPΔ3’ #1 cells were pre-treated with a pair of TALENs designed to induce DSB in the region adjacent to the 3’end of eGFPΔ3’ (the eGFP TALEN pair). The design and generation of the TALEN expression vectors are described in Materials and Methods. The eGFP TALEN pair was added 6 hours prior the transduction with AAV particles containing the donor template. As expected, adding the eGFP TALEN pair led to a significant increase of the basal frequency of specific HR events (from 3.6x10^-3^ ± 1.6x10^-3^ to 28x10^-3^ ± 6.5x10^-3^).

**Fig 5:**
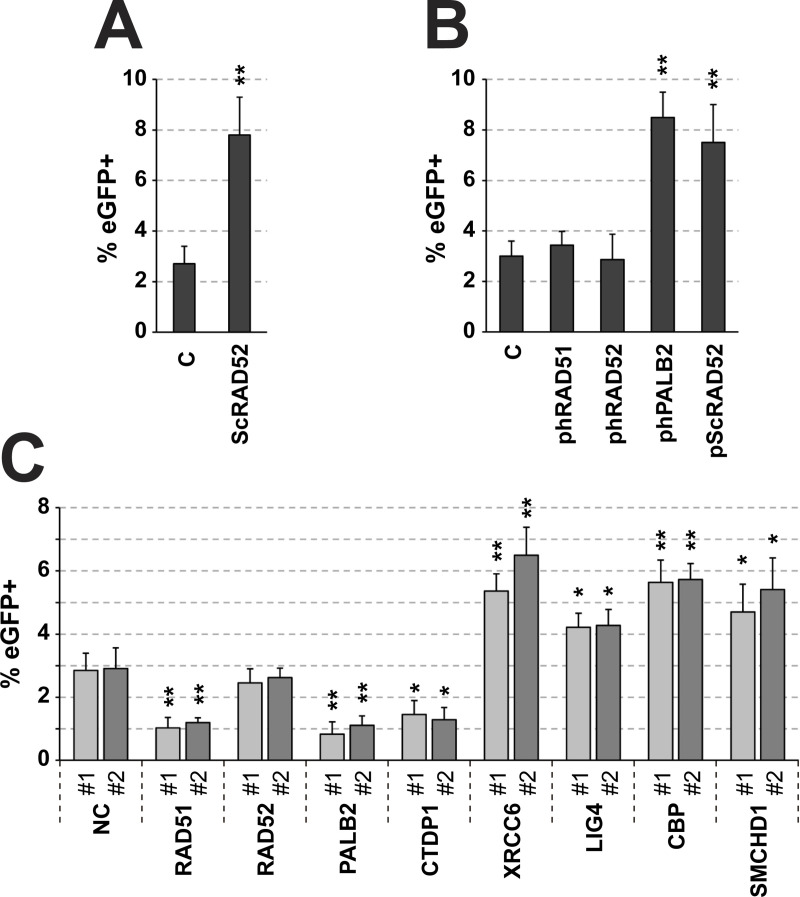
Validation of our eGFP-based assay for measuring the frequency of HR. (A) Effect of the addition of TAT-NLS-ScRAD52 on HR rates in our assay. Control cells (C) were treated with an equal volume of vehicle (see Materials and Methods). (B) Effect of the overexpression of plasmid-encoded HR promoters on HR rates. Empty pcDNA3 vector (vector) served as control. (C) Impact of various siRNAs targeting DNA repair-related genes on the frequency of HR. Two independent siRNAs were tested for each of the silenced genes. Two negative control siRNAs were used: NC#1 served as a negative control for all Silencer Select siRNAs and NC#2 served as a negative control for classical siRNAs (this information is available in **S3 Table**). Results are depicted as percentage of eGFP+ cells. Mean values with SD are shown (n = 3 independent experiments). *p < 0.05, **p < 0.01, ***p < 0.001. Raw data underlying these results and representative flow cytometry dot plots graphs showing the number of eGFP+ cells are available in **S2 Fig**.

First, we tested how adding TAT-NLS-ScRAD52 impacted the frequency of HR. RAD52 is an important HR protein that has the strongest effect on *Saccharomyces cerevisiae*. In this regard, the yeast protein, ScRAD52, has been demonstrated to be more effective than its human homologue in promoting HR [28]. The TAT-NLS-ScRAD52 fusion protein was generated as described in the Materials and Methods section. The TAT peptide made ScRAD52 cell permeable and the NLS targeted the protein to the nucleus. TAT-NLS-ScRAD52 was added to the HCT116-eGFPΔ3’ #1 reporter cell line 5 hours after the transfection of the eGFP TALEN vectors and 1 hour before the infection with the AAV particles containing the donor plasmid. As expected, the addition of TAT-NLS-ScRAD52 resulted in an approximate 3-fold increase in HR frequency **(Fig 5A)**. We also explored the effect of the overexpression of the following human genes known to promote HR: RAD51, RAD52, and PALB2 [28–30]. The HCT116-eGFPΔ3’ #1 reporter cells were transfected with expression plasmids encoding the different Flag-tagged HR promoters and 24 hours later they were transfected with the eGFP TALEN vectors. Six hours after the transfection of the TALEN vectors, the reporter cells were transduced with AAV particles containing the donor plasmid. As expected, the overexpression of PALB2 and ScRAD52 led to an increase in HR frequency ranging from approximately 2.5-fold increase in the case of ScRAD52 to 2.8-fold increase when PALB2 was overexpressed **(Fig 5B)**. It is of note that the plasmid encoded ScRAD52 increased HR frequency approximately as much as TAT-NLS-ScRAD52. In contrast, the overexpression of RAD51 and RAD52 had no significant effect on HR frequency. We comment on this result in the discussion section. The expression of the Flag-tagged proteins was confirmed by Western Blot **(S3 Fig)**.

Second, we explored the effect of various siRNAs targeting well-characterized DNA repair-related genes on the frequency of HR. In these assays, the HCT116-eGFPΔ3’ #1 reporter cells were transfected with different siRNAs and then treated with the eGFP TALEN and donor plasmid at the same times as those of the overexpression experiments described above. We used two siRNAs for each of the following genes: RAD51, RAD52, PALB2, CTDP1, XRCC6, LIG4, CBP and SMCHD1. As indicated above, RAD51, RAD52, and PALB2 play a major role in HR-mediated DNA repair [28–30], as does CTDP1 [31]. The efficacy of the siRNAs in gene silencing was validated by real-time PCR assay **(S3 Fig)**. As expected, the silencing of RAD51, PALB2 and CTDP1 led to a significant decrease in the HR rate **(Fig 5C)**. The silencing of RAD52 had no effect, which we comment on in the discussion section. The XRCC6 and LIG4 genes encode protein Ku70 and human ATP-dependent DNA ligase respectively, which are the components of the NHEJ repair pathway [32]. It has been reported that a decrease in NHEJ leads to a compensatory increase in HR. Accordingly, XRCC6- and LIG4-silenced reporter cells exhibited increased frequency of HR (**Fig 5C**). Similarly, reporter cells silenced for CBP and SMCHD1 expression exhibited increased frequency of HR. It is important to note that both CBP and SMCHD1 facilitate the recruitment of NHEJ factors at DSB sites, so their depletion was also expected to lead to an increase in HR rates [33, 34]. Of note, the silencing of XRCC6 and CBP led to the greatest increase in HR. Similar results were obtained between different siRNAs against the same gene in all cases.

## Discussion

In this study, we present the development and validation of a novel assay in which HR reconstitutes the full length eGFP from a 5’ and a 3’ truncated copies of eGFP.

The eGFPΔ3’ fragment, the HR substrate, consisted of a eGFP copy lacking the nucleotides encoding the last 24 amino acids, which are required for the protein’s fluorescence [21]. This transgene was integrated into the AAVS1 locus of HCT116 cells, which are commonly used for studying HR mechanisms [24–26]. Although AAVS1 is considered a well validated safe harbor in the human genome, it is important to recall that transgene integration at this locus disrupts the gene phosphatase 1 regulatory subunit 12C (PPP1R12C). As described above, the transgene contained a puromycin cassette preceded by a splice acceptor sequence in addition to the CMV promoter-driven eGFPΔ3’. As reported for other transgenes containing splice acceptor sites [35], the insertion of the puromycin cassette into the AAVS1 locus led to a downregulation of PPP1R12C mRNA levels **(S4 Fig)**. In this regard, it is important to mention that although the haploinsufficiency or complete inactivation of PPP1R12C does not lead to a discernible phenotype, the consequences of this disruption should be further investigated [27]. Another point we want to highlight is that the eGFPΔ3’ transgene was integrated as a single copy, and this is relevant when calculating the frequency of recombination because the appearance of a fluorescent signal in a cell is the result of a single HR event. Finally, it should be noted that the expression of the truncated eGFPΔ3’ copy could not be detected by western blot using an antibody against full length protein, suggesting the degradation of aberrant transcript and protein. Interestingly, the transgene has persisted over several passages (over 40) in our reporter cell line, which is likely due to the chromosomal stability of HCT116 [36].

The donor template consisted of a copy of eGFP lacking 42 bp at the 5’ end (including the start codon) and was delivered into the reporter cells via AAV particles. AAVs are on the rise as a powerful tool in gene therapy thanks to their lack of pathogenicity, wide range of cell tropism and long-term gene expression among other properties [37]. It is also well known that recombinant AAV vectors facilitate homologous recombination in mammalian cells at high efficiencies (even up to 10x10^-3^) and this is thought to be due, at least in part, to its single stranded nature [38]. The recombination frequency in our system was 3.6x10^-3^ ± 1.6x10^-3^, which falls within the range of values obtained by others using recombinant AAVs [39].

Finally, our study also validates the ability of our assay to identify factors affecting HR. Of note, all validation experiments were performed in the presence of a pair of TALENs designed to induce DSB in the region adjacent to the 3’ end of eGFPΔ3’. As expected, the addition of the eGFP TALEN pair led to a significant increase of the basal frequency of specific HR events (by approximately 8-fold), which helped improve the system.

ScRAD52 is more effective than its human homologue in promoting HR both in vitro and in vivo [28, 40, 41]. We observed that overexpression of ScRAD52 resulted in an approximate 3-fold increase in HR frequency when either the recombinant protein or the plasmid encoded protein were used. Similarly, the overexpression of PALB2 led to an increase in the HR frequency as previously reported by others using the DR-GFP reporter system [30]. In our assay neither RAD51 nor RAD52 had any significant effect on HR frequency. In this regard, it is important to mention that it has been previously reported that the overexpression of RAD51 does not have a significant effect on HR rates using DR-GFP system, while the overexpression of its dominant negative forms which are ATPase mutants results in a 8-fold decrease in HR frequency [42]. On the other hand, Kim et al. have shown that the overexpression of RAD51 and/or RAD52 reduces DSB-induced HR while enhances spontaneous HR [43]. The distinct results obtained by us and others may reflect differences in recombination substrate structures or different levels of overexpression.

The impact of siRNA-mediated silencing of RAD51, RAD52, PALB2, CTDP1, XRCC6, LIG4, CBP and SMCHD1 on the HR frequency that we report here is similar to that observed in previous genome wide HR screens and single gene approaches by others [31, 44–46]. Thus, as expected, RAD51, PALB2, and CTDP1 depletion resulted in a marked reduction of eGFP+ cells whereas depletion of NHEJ-related genes led to an increase in their number. This last result highlights the tight regulation of the balance between NHEJ and HR. In the case of RAD52, its silencing had a negligible effect on HR frequency. This apparently unexpected result has been reported by others, indicating that RAD52 is not essential to HR [44, 45, 47].

In conclusion, we have established a sensitive assay for measuring the efficiency of HR in the context of the DNA repair of an integrated transgene using an exogenous DNA template. This novel tool will be useful to accurately test the impact of continually emerging tools for HR-mediated genome editing, drugs, miRNAs and genes on HR and DNA repair. We anticipate that refining the precision of repair during in vitro culture will contribute to developing innovative gene therapy products.

## Supporting information

S1 Table Primers used in this study. Restriction enzymes sites are underlined in the primer sequence. The Flag-tag sequence is indicated in lower-case letters.
(DOCX)

S2 Table Control and eGFP MLPA probes. Lower-case non-italicized letters show sequences that do not match genomic DNA, but to which PCR primers are targeted; lower-case italicized letters indicate “stuffer” sequence; capital letters show unique sequence that targets genomic DNA.
(DOCX)

S3 Table siRNAs used in this study. Two types of siRNAs were used: Silencer Select (SS) siRNAs from Thermo Fisher Scientific and classical siRNAs from Merck. NC#2 siRNA has been extensively validated and its targeting sequence is 5’-GCAUUCACUUGGAUAGUAA-3’. The displayed siRNA location is relative to the beginning of the Ref Seq sequence.
(DOCX)

S1 Fig Experimental protocol. Self-explanatory graphical representation of the newly developed HR assay.
(EPS)

S2 Fig Analysis of flow cytometry data. (A) Representative flow cytometry dot plots graphs showing the gating strategy and the number of eGFP+ cells. Dead cells and debris were excluded based on scatter signals (in some experiments they were excluded based on propidium iodide fluorescence). HCT116-eGFPΔ3’ and HCT116-rec-eGFP cells were used as negative and positive controls, respectively. Cell treatments are indicated above the graphs. (B) Analysis of the raw data underlying the results in **Fig 5**. The number of eGFP+ cells out of 50,000 gated events and the formula used to calculate the percentage of eGFP+ cells are shown. The calculation takes into account transfection and transduction efficiencies which were determined for expression plasmids, siRNAs and AAVs as detailed in Materials and Methods. NC#1 and NC#2 siRNAs were used to calculate the transfection efficiency of SS siRNAs, and classical siRNAs, respectively.
(PDF)

S3 Fig Overexpression and silencing of HR related genes. (A) Representative Western blot analysis of total cell extracts from HCT116-eGFPΔ3’ cells 24 hours after transfection with the indicated plasmids using mouse monoclonal antibodies against Flag peptide or b-actin (loading control). Empty vector corresponds to pcDNA3 vector. (B) The efficacy of the indicated siRNAs in gene silencing was validated by a SYBR Green based real-time PCR assay using the primers listed in **S1 Table**. Results are depicted as fold change relative to HCT116-eGFPΔ3’ cells treated with negative control siRNAs (set as 1). Two negative control siRNAs were used: NC#1 served as a negative control for all Silencer Select siRNAs and NC#2 served as a negative control for classical siRNAs (this information is available in **S3 Table**). Mean values with SD are shown (n = 3 independent experiments). *p < 0.05, **p < 0.01, ***p < 0.001.
(EPS)

S4 Fig Expression of PPP1R12C in HCT116-eGFPΔ3’ cells. Transcript levels were measured by SYBR Green based real-time PCR assay using a forward primer located in PPP1R12C exon 1 and a reverse primer located in PPP1R12C exon 2 (**S1 Table**). Results are depicted as fold change relative to parental HCT116 cells (set as 1). Mean values with SD are shown (n = 3 independent experiments). *p < 0.05, **p < 0.01, ***p < 0.001.
(EPS)

S1 Raw images (PDF)
