# Supplementary material for: Novel fluorescent-based reporter cell line engineered for monitoring homologous recombination events
Source: PLoS One. 2021 Apr 30;16(4):e0237413. doi: 10.1371/journal.pone.0237413 (PMC8087102; doi:10.1371/journal.pone.0237413)
Supplement: S1 Table — Restriction enzymes sites are underlined in the primer sequence. The Flag-tag sequence is indicated in lower-case letters. (DOCX) [file pone.0237413.s001.docx]

**S1 Table**

| **Name** | **Sequence (5’-3’)** | **Restriction site** |
| --- | --- | --- |
| 1F | ATTGCGGCCGCACGAATTCGCCCTTTGCTTTC | NotI |
| 1R | ATCGTCGACGGCGTTACTATGGGAACATACG | SalI |
| 2F | ATTGTCGACTGTGGATAACCGTATTACCG | SalI |
| 2R | ATTGAATTCTTCTCGTTGGGGTCTTTGC | EcoRI |
| 3F | ATTGAATTCACTAGGGACAGGATTGGTGAC | EcoRI |
| 3R | AATGCGGCCGCAGGACGAGAAACACAGCCCC | NotI |
| 4F | AATAGGGCCCATCCTGGTCGAGCTG | PspOMI |
| 4R | TAAGTCGACTTAAGATACATTGATGAGTTTG | SalI |
| ScRAD52_F1 | TGTAAGCTTGAAGAAGAAGAGGAAGGTGTCCATGAATGAAATTATGGATATGGATG | HindIII |
| ScRAD52_R1 | ATTCTCGAGTCAAGTAGGCTTGCGTGCATG | XhoI |
| ScRAD52_F2 | ATTGAATTCGCCACCatggattataaagatgatgatgataaagtaccaggaAATGAAATTATGGATATGGATGAGAAG | EcoRI |
| ScRAD52_R2 | ATTCTCGAGTCAAGTAGGCTTGCGTGCATGCAG | XhoI |
| hRAD51_F | TAAGGTACCGCCACCatggactacaaggacgacgatgacaaggtaccaggaGCGATGCAGATGCAGTTGGAAG | KpnI |
| hRAD51_R | ATTGAATTCAATCTTTGGCGTCCCCTACG | EcoRI |
| hRAD52_F | TAAGGTACCGCCACCatggactacaaggacgacgatgacaaggtaccaggaTCTGGGACTGAGGAAGCAATTC | KpnI |
| hRAD52_R | ATTGAATTCTTAAGATGGATCATATTTCCTTTTCTTC | EcoRI |
| P1F | GACCACTTTGAGCTCTACTG | none |
| P1R | CCGCATGTTAGAAGACTTCC | none |
| P2F | CGACCACTACCAGCAGAAC | none |
| P2R | CCAAGTGGTTGATAAACCCAC | none |
| P3F | TAGTGAACCGTCAGATCCGC | none |
| P3R | CAGGTTCAGGGGGAGGTGTG | none |
| hSDHA_F | CATGGTGCGCAGGCGCGATGT | none |
| hSDHA_R | CTGCTCCTCAGGACAACCC | none |
| T1F | ACGTAAACGGCCACAAGTTC | none |
| T1R | AAGTCGTGCTGCTTCATGTG | none |
| T2F | TATATCATGGCCGACAAGCA | none |
| T2R | ACTGGGTGCTCAGGTAGTGG | none |
| C1F | CACTTCCAGGAGTCGCTGT | none |
| C1R | CCTTTACCCGGTGCTACACA | none |
| C2F | GCCTCACCTGGCGATATCTA | none |
| C2R | CGTGTCCCTGGATCTGTTTT | none |
| CBP_F | CAACCCCAAAAGAGCCAAAC | none |
| CBP_R | GGTTCCCACTGTTTAAAAGGC | none |
| CTDP1_F | TCAGTCCCGTGTAGCCTC | none |
| CTDP1_R | CATCCTTCCAACCTCACCAG | none |
| LIG4_F | GCTGGGATTCTCTGGTTCAC | none |
| LIG4_R | CTGCAAAAGGAACGTGAGATG | none |
| PALB2_F | GCATAAACATTCCGTCGAACAG | none |
| PALB2_R | CGGCTCTTTCCCAAAACATG | none |
| RAD51_F | GTGGTAGCTCAAGTGGATGG | none |
| RAD51_R | GGGAGAGTCGTAGATTTTGCAG | none |
| RAD52_F | GGTTATGGTGTTAGTGAGGGC | none |
| RAD52_R | GATCTCAGGTAGTCTTTGTCCAG | none |
| SMCHD1_F | AAGTCCTGCCTAATCAACCTG | none |
| SMCHD1_R | CCAGTATGGTTGTCGTAGTCATC | none |
| XRCC6_F | AGTGACAGCTTTGAGAACCC | none |
| XRCC6_R | GGAGCCCAGTCTTTTATTCATTG | none |
| PPP1R12C_F | CAACGCCGACGGTATCAG | none |
| PPP1R12C_R | CTCGTTGTCTGCCTGGTTC | none |
| HPRT1_F | CCTGGCGTCGTGATTAGTGATGAT | none |
| HPRT1_R | AGCAAGACGTTCAGTCCTGTCCAT | none |
| ACTB_F | ACCAACTGGGACGACATGGAGAAA | none |
| ACTB_R | TAGCACAGCCTGGATAGCAACGTA | none |
