# Supplementary material for: Novel fluorescent-based reporter cell line engineered for monitoring homologous recombination events
Source: PLoS One. 2021 Apr 30;16(4):e0237413. doi: 10.1371/journal.pone.0237413 (PMC8087102; doi:10.1371/journal.pone.0237413)
Supplement: S2 Table — Lower-case non-italicized letters show sequences that do not match genomic DNA, but to which PCR primers are targeted; lower-case italicized letters indicate “stuffer” sequence; capital letters show unique sequence that targets genomic DNA. (DOCX) [file pone.0237413.s002.docx]

**S2 Table**

| **Probe name** | **Probe length** | **Target** | **Sequence** |
| --- | --- | --- | --- |
| eGFP1_5’ | 66 | eGFP | 5’-gggttccctaagggttgga *cgctactactattagtagaattgat* CGCACCATCTTCTTCAAGGACG-3’ |
| eGFP1_3’ | 66 | eGFP | 5’-ACGGCAACTACAAGACCCGCG *ctaatggtcaaactaaatctac* tctagattggatcttgctggcgc-3’ |
| eGFP2_5’ | 84 | eGFP | 5’-gggttccctaagggttgga *cgctactactattagtagaattgatgccaccttttcagctcgcg* AAGTTCATCTGCACCACCGGC-3’ |
| eGFP2_3’ | 84 | eGFP | 5’-AAGCTGCCCGTGCCCTGGCCC *accatttgcgaaatgtatctaatggtcaaactaaatctac* tctagattggatcttgctggcgc-3’ |
| C1_5’ | 46 | chr22:30,069,316 | 5’-gggttccctaagggttgga *cgctac* GGCCCAGATCACCGAGGAGGA-3’ |
| C1_3’ | 47 | chr22:30,069,316 | 5’-GGCAAAACTTCTGGCCCAGAAG *ac* tctagattggatcttgctggcgc-3’ |
| C2_5’ | 55 | chr1:156,105,841 | 5’-gggttccctaagggttgga *cgctactactat* CAGCTGGACGAGTACCAGGAGCTT-3’ |
| C2_3’ | 56 | chr1:156,105,841 | 5’-CTGGACATCAAGCTGGCCCTG *aactaaatctac* tctagattggatcttgctggcgc-3’ |
| C3_5’ | 72 | chr17:3,397,683 | 5’-gggttccctaagggttgga *cgctactactattagtagaattgatg* TCCCTGCGCCATTGAGGTCTATAAAAT-3’ |
| C3_3’ | 72 | chr17:3,397,683 | 5’-TATAGAGAAAGTTGATTACCCCCGGGATG *aatggtcaaactaaatctac* tctagattggatcttgctggcgc-3’ |
| C4_5’ | 86 | chr11:14,515,205-14,515,256 | 5’-gggttccctaagggttgga *cgctactactattagtagaattgatgccaccttttcagctcgcg* TGCATGTTTGGAGCATCGACACA-3’ |
| C4_3’ | 86 | chr11:14,515,205-14,515,256 | 5’-GCTATGTTAGAAGAAATGCTGTTTTGGCC *tgcgaaatgtatctaatggtcaaactaaatctac* tctagattggatcttgctggcgc-3’ |
| C5_5’ | 62 | chr2:109,545,837 | 5’-gggttccctaagggttgga.*cgctactactattagtagaat.*AGTCCTGTGGCTACGGCACCAA-3’ |
| C5_3’ | 62 | chr2:109,545,837 | 5’-AGACGAGGACTACGGCTGCGTC.*ggtcaaactaaatctac.*tctagattggatcttgctggcgc-3’ |
| C6_5’ | 59 | chr7:129,409,814 | 5’-gggttccctaagggttgga *cgctactactattagt* GCCCAGGAAGGAACCACTCTTAGT-3’ |
| C6_3’ | 58 | chr7:129,409,814 | 5’-GACCTGTGGAAGGTTAGGAGACCC *actaaatctac* tctagattggatcttgctggcgc-3’ |
| C7_5’ | 76 | chr11:569,809 | 5’-gggttccctaagggttgga *cgctactactattagtagaattgatgccaccttttc* GGGAAGGTGCCCAGAGGATCA-3’ |
| C7_3’ | 76 | chr11:569,809 | 5’-CGGAGCCACTCGGTTCTATTGG *ttgcgaaatgtatctaatggtcaaactaaat* tctagattggatcttgctggcgc-3’ |
