# Supplementary material for: Novel fluorescent-based reporter cell line engineered for monitoring homologous recombination events
Source: PLoS One. 2021 Apr 30;16(4):e0237413. doi: 10.1371/journal.pone.0237413 (PMC8087102; doi:10.1371/journal.pone.0237413)
Supplement: S3 Table — Two types of siRNAs were used: Silencer Select (SS) siRNAs from Thermo Fisher Scientific and classical siRNAs from Merck. NC#2 siRNA has been extensively validated and its targeting sequence is 5’-GCAUUCACUUGGAUAGUAA-3’. The displayed siRNA location is relative to the beginning of the Ref Seq sequence. (DOCX) [file pone.0237413.s003.docx]

**S3 Table**

| **Gene symbol** | **Full Gene Name** | **Gene ID** | **Ref Seq** | **siRNA identification** | **targeted exon(s)** | **siRNA location** |
| --- | --- | --- | --- | --- | --- | --- |
| **CREBBP/CBP** | CREB binding protein | 1387 | NM_001079846 | CBP#1: SS siRNA s3497 | 18,19 | 3782 |
|  |  |  |  | CBP#2: SS siRNA s3495 | 24 | 4257 |
| **CTDP1** | CTD phosphatase subunit 1 | 9150 | NM_001202504 | CTDP1#1: SS siRNA s17492 | 7 | 665 |
|  |  |  |  | CTDP1#2: SS siRNA s17493 | 8 | 1722 |
| **LIG4** | DNA ligase 4 | 3981 | NM_002312 | LIG4#1: SS siRNA s8181 | 2 | 1925 |
|  |  |  |  | LIG4#2: SS siRNA s8179 | 2 | 2546 |
| **PALB2** | Partner and localizer of BRCA2 | 79728 | NM_024675 | PALB2#1: SS siRNA s536268 | 5,6 | 2716 |
|  |  |  |  | PALB2#2: SS siRNA s36199 | 13 | 3627 |
| **RAD51** | RAD51 recombinase | 5888 | NM_133487 | RAD51#1: SS siRNA s11734 | 7,8 | 943 |
|  |  |  |  | RAD51#2: SS siRNA s11735 | 9 | 1095 |
| **RAD52** | RAD52 homolog | 5893 | NM_001297419 | RAD52#1: SS siRNA s532174 | 6 | 657 |
|  |  |  |  | RAD52#2: SS siRNA s258097 | 7 | 750 |
| **SMCHD1** | structural maintenance of chromosomes flexible hinge domain containing 1 | 23347 | NM_015295 | SMCHD1#1: SS siRNA s23612 | 10 | 1356 |
|  |  |  |  | SMCHD1#2: SS siRNA s23613 | 20 | 2690 |
| **XRCC6** | X-ray repair cross complementing 6 | 2547 | NM_001469 | XRCC6#1: classical siRNA | 3 | 463 |
|  |  |  |  | XRCC6#2: classical siRNA | 2 | 324 |
| **SiSel_NC1** | Silencer Select Negative Control #1 siRNA |  |  | NC#1: SS siRNA s813 |  |  |
| **D0_NC2** | Negative Control #2 siRNA |  |  | NC#2: classical siRNA |  |  |
