# Supplementary material for: Novel fluorescent-based reporter cell line engineered for monitoring homologous recombination events
Source: PLoS One. 2021 Apr 30;16(4):e0237413. doi: 10.1371/journal.pone.0237413 (PMC8087102; doi:10.1371/journal.pone.0237413)
Supplement: S2 Fig — (A) Representative flow cytometry dot plots graphs showing the gating strategy and the number of eGFP+ cells. Dead cells and debris were excluded based on scatter signals (in some experiments they were excluded based on propidium iodide fluorescence). HCT116-eGFPΔ3’ and HCT116-rec-eGFP cells were used as negative and positive controls, respectively. Cell treatments are indicated above the graphs. (B) Analysis of the raw data underlying the results in Fig 5. The number of eGFP+ cells out of 50,000 gated events and the formula used to calculate the percentage of eGFP+ cells are shown. The calculation takes into account transfection and transduction efficiencies which were determined for expression plasmids, siRNAs and AAVs as detailed in Materials and Methods. NC#1 and NC#2 siRNAs were used to calculate the transfection efficiency of SS siRNAs, and classical siRNAs, respectively. (PDF) [file pone.0237413.s005.pdf]

A

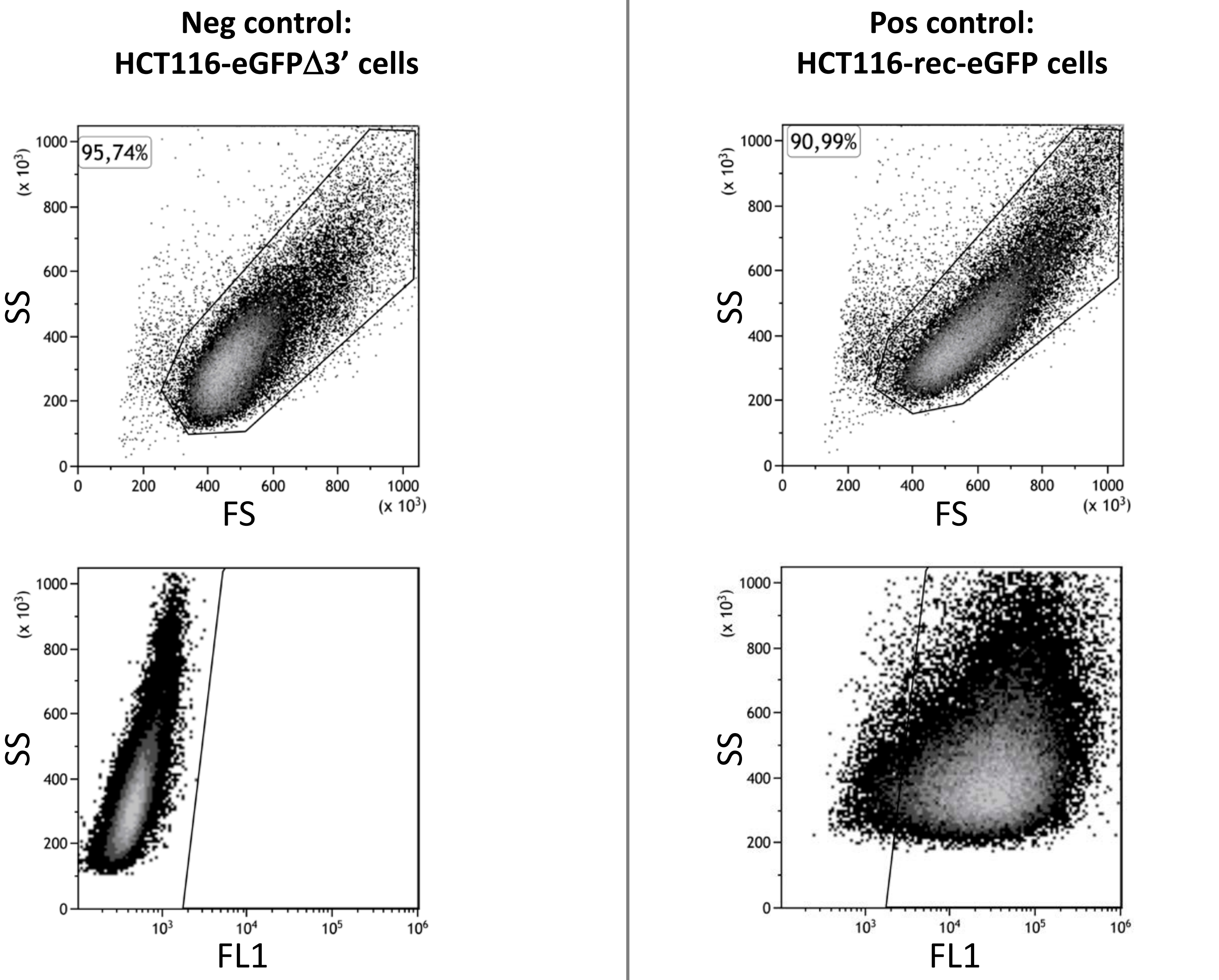

Neg control:
HCT116-eGFPΔ3' cells
Pos control:
HCT116-rec-eGFP cells
95,74%
90,99%
SS
FS
FL1
(x 10³)

HCT116-eGFPΔ3' cells
siRNA NC#1

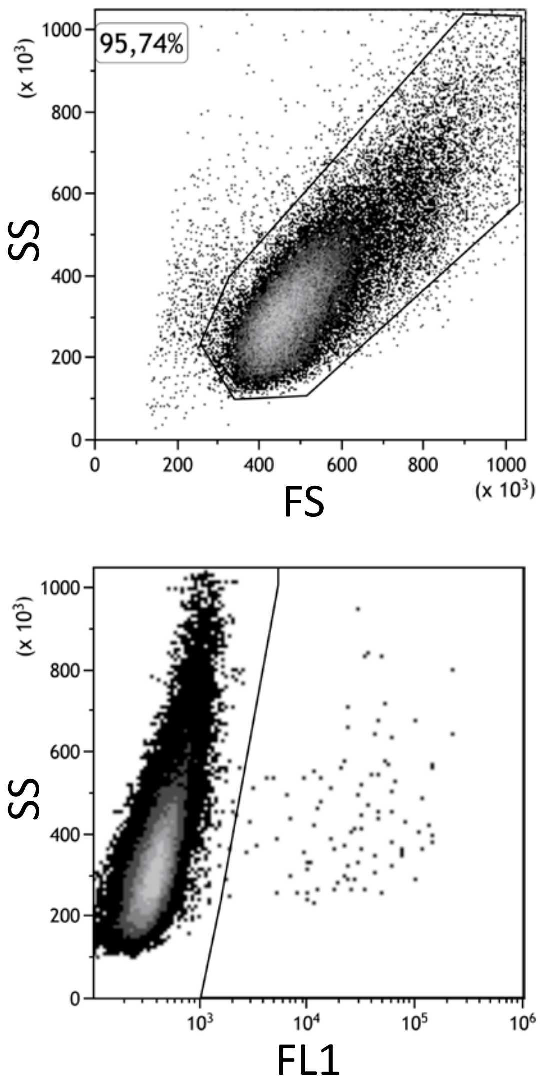

90 eGF+ cells

HCT116-eGFPΔ3' cells
siRNA CBP#2

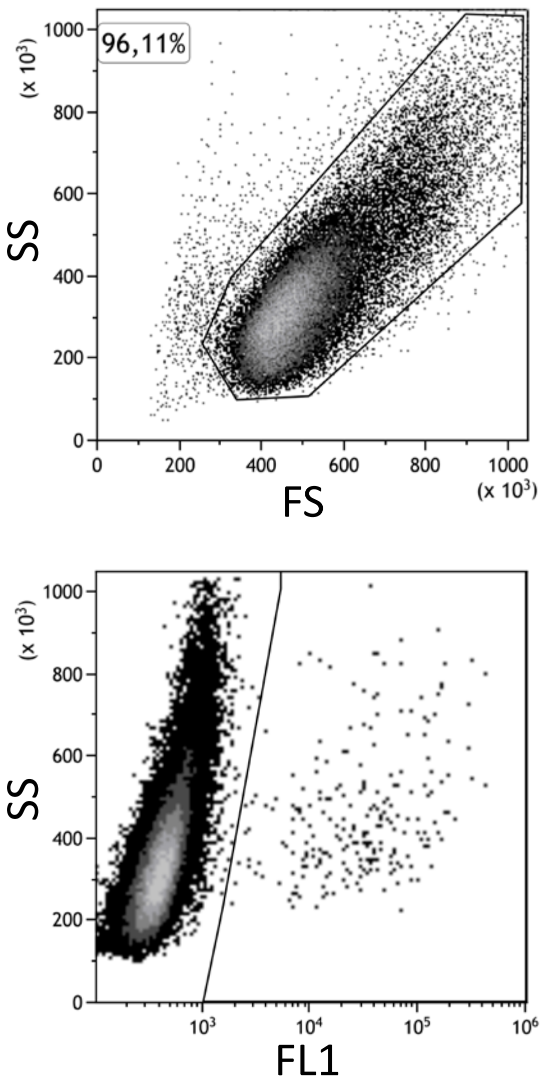

253 eGF+ cells

HCT116-eGFPΔ3' cells
siRNA SMCHD1#1

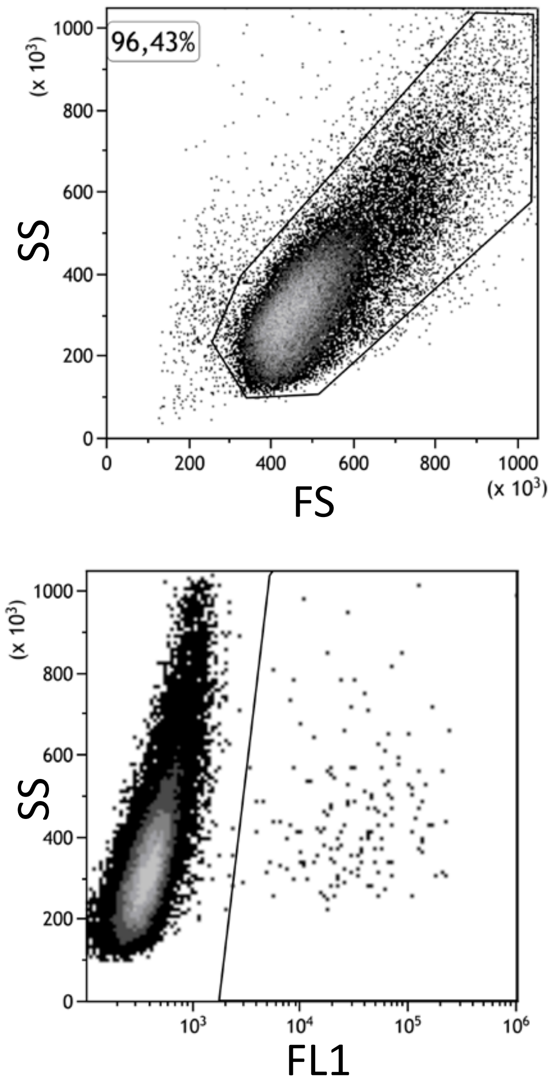

154 eGF+ cells

B

$$\% \, eGFP \, positive \, cells = \frac{\left(\frac{number \, of \, eGFP \, positive \, cells}{50{,}000}\right) \times 100}{\frac{\% \, transfection \, effcy \, (plasmid \, or \, siRNA)}{100} \times \frac{\% \, transfection \, effcy \, (TALEN)}{100} \times \frac{\% \, transduction \, effcy \, (AAV)}{100}}$$

**Figure A**	**number of EGFP+ cells/50.000**			**% EGFP+ cells**						
**treatment**	**Exp#1**	**Exp#2**	**Exp#3**	**Exp#1**	**Exp#2**	**Exp#3**	**Mean**	**SD**	**t student**	
Control	185	88	127	3,422	1,971	2,739	2,711	0,726		
ScRAD52	498	278	365	9,239	6,242	7,892	7,791	1,501	0,006	**
**Figure 5B**	**number of EGFP+ cells/50.000**			**% EGFP+ cells**						
**plasmid**	**Exp#1**	**Exp#2**	**Exp#3**	**Exp#1**	**Exp#2**	**Exp#3**	**Mean**	**SD**	**t student**	
pcDNA3	152	94	134	3,435	2,284	3,221	2,98	0,612		
phRAD51	175	114	148	3,906	2,846	3,554	3,435	0,54	0,389	
phRAD52	151	68	147	3,378	1,706	3,529	2,871	1,012	0,881	
phPALB2	409	293	376	9,248	7,232	9,041	8,507	1,109	0,002	**
pScRAD52	270	363	300	6,106	9,070	7,291	7,489	1,492	0,008	**
**Figure 5C**	**number of EGFP+ cells/50.000**			**% EGFP+ cells**						
**siRNA**	**Exp#1**	**Exp#2**	**Exp#3**	**Exp#1**	**Exp#2**	**Exp#3**	**Mean**	**SD**	**t student**	
NC#1	145	90	123	3,28	2,246	3,02	2,849	0,538		
NC#2	138	86	143	3,109	2,18	3,432	2,907	0,650	0,911	
RAD51#1	50	26	52	1,132	0,658	1,288	1,026	0,328	0,007	**
RAD51#2	59	49	42	1,334	1,223	1,034	1,197	0,152	0,007	**
RAD52#1	105	82	119	2,364	2,051	2,938	2,451	0,450	0,381	
RAD52#2	129	93	107	2,912	2,322	2,631	2,622	0,295	0,557	
PALB2#1	50	15	39	1,126	0,386	0,969	0,827	0,390	0,006	**
PALB2#2	61	32	47	1,375	0,796	1,164	1,112	0,293	0,008	**
CTDP1#1	49	52	79	1,116	1,295	1,951	1,454	0,440	0,025	*
CTDP1#2	76	38	48	1,719	0,957	1,172	1,283	0,393	0,015	*
XRCC6#1	236	191	246	5,339	4,823	5,912	5,358	0,545	0,008	**
XRCC6#2	330	228	261	7,46	5,746	6,288	6,498	0,876	0,005	**

LIG4#1	206	152	170	4,667	3,794	4,193	4,218	0,437	0,027	*
LIG4#2	187	193	154	4,225	4,809	3,791	4,275	0,511	0,029	*
CBP#1	279	228	200	6,313	5,696	4,920	5,643	0,698	0,005	**
CBP#2	238	253	223	5,391	6,306	5,49	5,729	0,502	0,003	**
SMCHD1#1	207	154	227	4,673	3,844	5,593	4,703	0,875	0,035	*
SMCHD1#2	276	227	176	6,249	5,66	4,336	5,415	0,980	0,017	*

	Exp#1	Exp#2	Exp#3
Transfection effcy pcDNA3	82,0	92,0	90,0
Transfection effcy phRAD51	83,0	90,0	90,0
Transfection effcy phRAD52	83,0	90,0	90,0
Transfection effcy phPALB2	82,0	91,0	90,0
Transfection effcy pScRAD52	82,0	90,0	89,0
Transfection effcy (SS siRNA NC#1)	82,0	90,0	88,0
Transfection effcy (Classical siRNA NC#2)	82,0	89,0	90,0
Transfection effcy (TALEN)	83,0	89,0	88,0
Transduction effcy (AAV)	13,0	10,0	10,5
