# Supplementary material for: Novel fluorescent-based reporter cell line engineered for monitoring homologous recombination events
Source: PLoS One. 2021 Apr 30;16(4):e0237413. doi: 10.1371/journal.pone.0237413 (PMC8087102; doi:10.1371/journal.pone.0237413)

Fig1B_P1F-P1R
GEL DOC

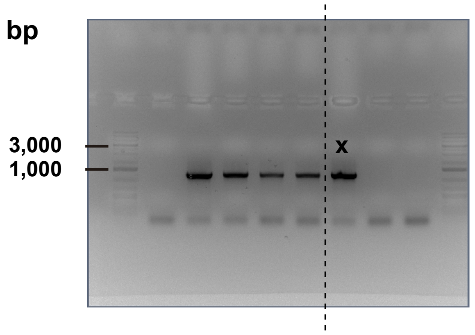

Fig1B_P2F-P2R
GEL DOC

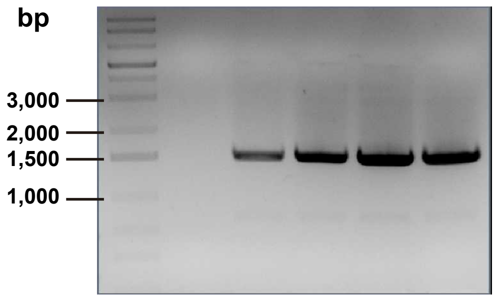

Fig1B_SDHA
GEL DOC

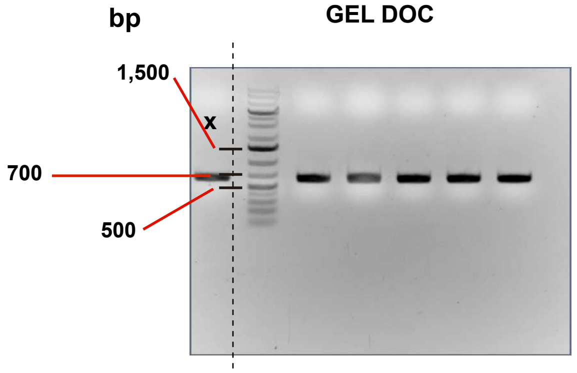

Fig1B_P1F-P2R
GEL DOC

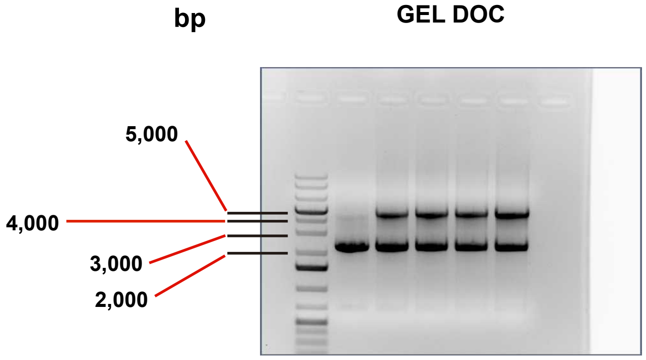

Fig4C_P3F-P3R
GEL DOC

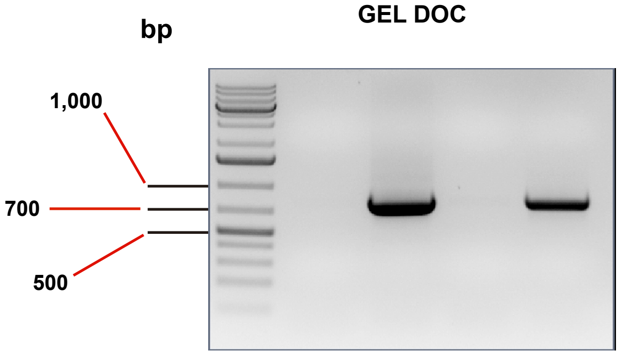

Fig4C_SDHA
GEL DOC

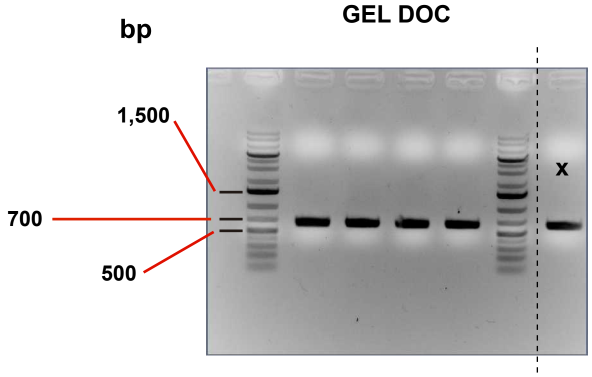

Fig4D_EGFP
SCAN

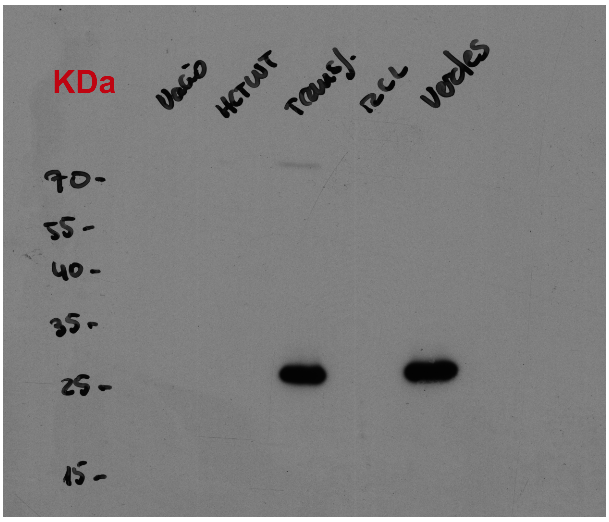

Fig4D_ACTIN
SCAN

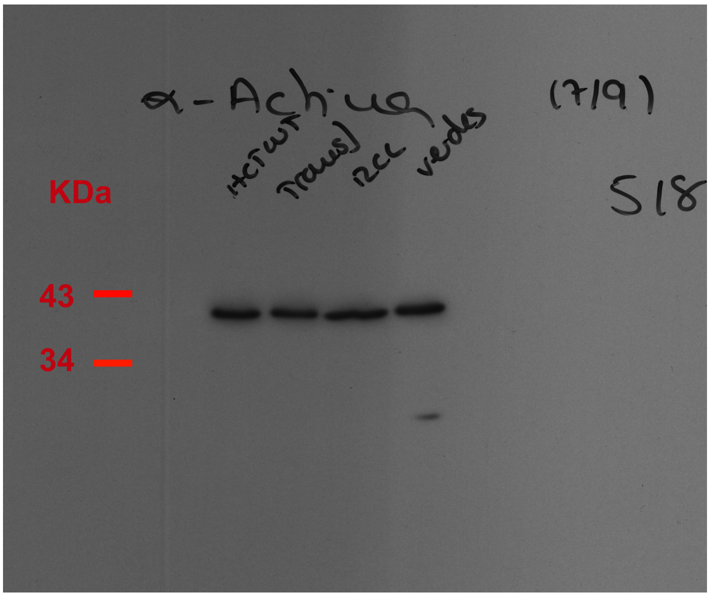

S2FigA_Flag
SCAN

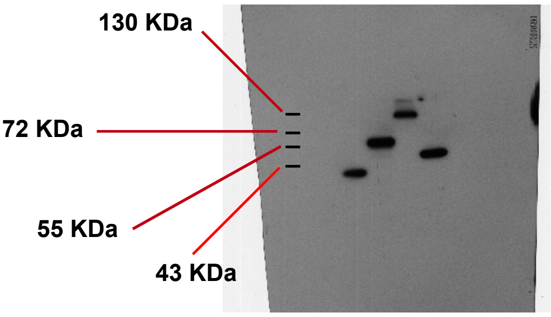

S2FigA_actin
SCAN

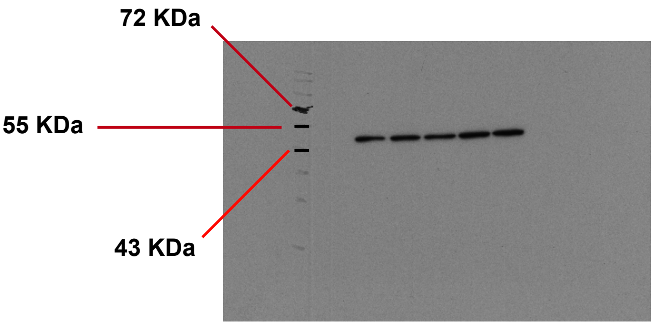

Supplement: S1 Raw images — (PDF) [file pone.0237413.s008.pdf]
